# Identification of a *Saltol*-Independent Salinity Tolerance Polymorphism in Rice Mekong Delta Landraces and Characterization of a Promising Line, Doc Phung

**DOI:** 10.1186/s12284-022-00613-0

**Published:** 2022-12-18

**Authors:** Tam Thanh Nguyen, Maria Stefanie Dwiyanti, Shuntaro Sakaguchi, Yohei Koide, Dung Viet Le, Toshihiro Watanabe, Yuji Kishima

**Affiliations:** 1 Research Faculty of Agriculture, Hokkaido University, Sapporo, 060-8589 Japan; 2 Mekong Delta Development Research Institute, Can Tho University, Campus 2 3-2 Street, Can Tho, Vietnam; 3 College of Agriculture, Can Tho University, Campus 2 3-2 Street, Can Tho, Vietnam

**Keywords:** Mekong Delta, Salinity tolerance, Rice, Doc Phung, GWAS (genome-wide association study), SES (standard evaluation system), Mineral concentrations, *OsPLGG1*

## Abstract

The Mekong Delta River in Vietnam is facing salinity intrusion caused by climate change and sea-level rise that is severely affecting rice cultivation. Here, we evaluated salinity responses of 97 rice accessions (79 landraces and 18 improved accessions) from the Mekong Delta population by adding 100 mM NaCl to the nutrient solution for up to 20 days. We observed a wide distribution in salinity tolerance/sensitivity, with two major peaks across the 97 accessions when using the standard evaluation system (SES) developed by the International Rice Research Institute. SES scores revealed strong negative correlations (ranging from − 0.68 to − 0.83) with other phenotypic indices, such as shoot elongation length, root elongation length, shoot dry weight, and root dry weight. Mineral concentrations of Na^+^ in roots, stems, and leaves and Ca^2+^ in roots and stems were positively correlated with SES scores, suggesting that tolerant accessions lower their cation exchange capacity in the root cell wall. The salinity tolerance of Mekong Delta accessions was independent from the previously described salinity tolerance–related locus *Saltol*, which encodes an HKT1-type transporter in the salinity-tolerant cultivars Nona Bokra and Pokkali. Indeed, genome-wide association studies using SES scores and shoot dry weight ratios of the 79 accessions as traits identified a single common peak located on chromosome 1. This SNP did not form a linkage group with other nearby SNPs and mapped to the 3′ untranslated region of gene LOC_Os01g32830, over 6.5 Mb away from the *Saltol* locus. LOC_Os01g32830 encodes chloroplast glycolate/glycerate translocator 1 (*OsPLGG1*), which is responsible for photorespiration and growth. SES and shoot dry weight ratios differed significantly between the two possible haplotypes at the causal SNP. Through these analyses, we characterize Doc Phung, one of the most salinity-tolerant varieties in the Mekong Delta population and a promising new genetic resource.

**Supplementary Information:**

The online version contains supplementary material available at 10.1186/s12284-022-00613-0.

## Background

Rice (*Oryza sativa* L.) is a main staple food source for about 90% of Asian people and is grown in areas with easy access to water. One of greatest threats to rice production is salinity stress (Maas and Grattan 1999). In coastal and/or acid and semi-arid areas, the effect of salinity conditions is a major issue for rice production. In particular, about 500,000 ha around coastal areas in the Mekong Delta/River are predicted to become affected by salinity intrusion caused by climate change and the rise of sea levels by 2050, with this number increasing tenfold when considering the worst case scenario of combined climate change, sea-level rise, and low river flows (Kontgis et al. 2019). Salinity intrusion from the sea can impose salinity stress during the dry season, when saltwater seeps into the ground freshwater (Nhung et al. 2019). The use of salinity-tolerant varieties is seen as a more efficient and effective mitigating measure than farming practices or salinity management facilities such as dikes, pumping stations, and sluices (Akbar et al. 1972).

Rice is generally sensitive to high-salinity conditions, especially at the seedling stage (Batayeva et al. 2018; De Leon et al. 2016; Kumari et al. 2009; Lutts et al. 1995; Mohammadi-Nejad et al. 2010; Puram et al. 2017; Tian et al. 2011). Much effort has been made to screen and select salinity-tolerant rice varieties (De Leon et al. 2017; Ganie et al. 2019; Hariadi et al. 2015; Lee et al. 2003), such as the high-salinity-tolerant ‘Nona Bokra’ and ‘Pokkali’, which have greatly contributed to salinity-tolerant rice breeding (Ren et al. 2005; Thomson et al. 2010). These *indica* strains are landraces from the Indian and Sri Lanka coastal regions, respectively. Their salinity-tolerance may have been achieved by adapting to high-salinity soils (Emon et al. 2015; Gregorio and Senadhira 1993). The *Saltol* locus defined a strong-effect quantitative trait locus (QTL) associated with this salinity-tolerance and was mapped to chromosome 1 (Bonilla et al. 2002; Xie et al. 2000). The *Saltol* locus was later shown to encode a HIGH-AFFINITY K^+^ TRANSPORTER1 (HKT1)-type sodium (Na^+^) transporter (Ren et al. 2005). However, the biological response to salt is extremely complex, and not all salinity-tolerance can be explained by the *Saltol* locus. In fact, other salinity-tolerant landrace varieties from Bangladesh and Saudi Arabia have a *Saltol* haplotype distinct from that of Nona Bokra or Pokkali (Bimpong et al. 2014; Rahman et al. 2017, 2019). Several QTL analyses have revealed various mechanisms involved in rice salinity-tolerance (Singh et al. 2021).

The mechanisms related to salinity-tolerance of rice can broadly be divided into three categories (Marschner 2012): Na^+^ exclusion, osmotic stress regulation, and tissue tolerance to Na^+^ accumulation (Munns and Tester 2008). Na^+^ absorbed by the roots is transported from the xylem to the shoots. Na^+^ accumulation in the shoots is negatively correlated with salinity-tolerance, as excess Na^+^ levels in cells have a detrimental effect on plant growth (Faiyue et al. 2012; Krishnamurthy et al. 2011; Platten et al. 2013). In the context of Na^+^ exclusion, the salinity-resistant *Saltol* haplotype, such as that harbored by Nona Bokra and Pokkali, rely on the activity of OsHKT1;5, a Na^+^ ion transporter that can avoid the accumulation of Na^+^ in shoots (Platten et al. 2013; Ren et al. 2005). Osmotic regulation modulates stomatal conductance before too much Na^+^ accumulates in shoots by reducing water evaporation and, thus, the flow of water from roots to shoots, resulting in salinity-tolerance (Rios et al. 2017; Termaat et al. 1985). Tissue tolerance refers to the mechanism that prevents toxic Na^+^ levels from accumulating in cells (Davenport et al. 2005). Several known mechanisms prevent Na^+^ from being released into the cytoplasm by sequestration of Na^+^ into vacuoles or by releasing Na^+^ into the soil (Anil et al. 2007; Bassil et al. 2012; Kader et al. 2007). Under saline conditions, concentrations for the cation Na^+^ and the anion Cl^–^ increase in all plant organs, while the absorption of K^+^, Ca^2+^, Mg^2+^, and Mn^2+^ decrease (Grattan and Grieve 1999). Limiting the uptake of these essential ions negatively affects plant growth (Kumar et al. 2015).

In this study, we investigated the salinity-tolerance and physiological characteristics of a diverse panel of landrace rice accessions cultivated in the Mekong Delta region (Tam et al. 2019). We focused on the rice landrace variety ‘Doc Phung’, known to be particularly resistant to salt in the Mekong Delta (Ho et al. 2018; Le 1999; Tin et al. 2021). Based on the genomic structure of these Mekong Delta populations and GWAS results, we explored and resolved the relationship between the degree of salinity-tolerance, physiological characteristics, and genomic structure. Analysis of whole-genome sequences provided evidence that Doc Phung carries a *Saltol* allele different from that of Nona Bokra or Pokkali and that the causative region of salinity-tolerance might have enabled the adaptation to the coastal areas of the Mekong Delta.

## Materials and Methods

### Plant Materials

Ninety-seven rice accessions consisting of 79 landraces and 18 improved accessions were selected from the Genebank of Mekong Delta Development Research Institute (MDI), Can Tho University, Vietnam (Additional file 2: Table S1) (Tam et al. 2019). The group of accessions exhibited no strong population structure bias according to our previous analyses (Tam et al. 2019). ‘Nipponbare’ and ‘Pokkali’ were added as sensitive and tolerant cultivar controls, respectively, for salinity stress. However, as Pokkali supplied by the International Rice Research Institute (IRRI) did not show the expected high tolerance in our assays, we stopped using this material.

### Screening for Salinity Tolerance

Seeds were treated with disinfectant (20% [v/v] solution of Tekurido C, Kumiai Chemical Industry Co., LTD) for 15 min for surface sterilization. Seeds were then washed with tap water three times and with distilled water two times. Surface-sterilized seeds were placed into petri dishes and germinated in dark conditions at 25 °C. Three days after germination, seedlings were transplanted to net sheets floating on distilled water in a 40-L container at 20–25 °C in the greenhouse (Additional file 1: Fig. S1). Four days after transfer (DAT), water was replaced with standard nutrient solution: 1.12 mM NH_4_NO_3_, 0.32 mM NaH_2_PO_4_, 0.19 mM K_2_SO_4_, 0.38 mM KCl, 1.25 mM CaCl_2_, 0.82 mM MgSO_4_, 35.8 µM Fe-EDTA, 9.1 µM Mn_2_SO_4_, 3.06 µM ZnSO_4_, 0.16 µM CuSO_4_, 46.2 µM H_3_BO_3_, and 0.052 µM (NH_4_)_6_Mo_7_O_24_. The nutrient solution was replaced every 3 days, and the pH was adjusted to 5.0 every day with 1 M NaOH or 0.5 M HCl, as needed. At 15 DAT, the salinity stress treatment was started by adding 100 mM NaCl to the nutrient solution. Immediately before treatment, the shoot and root lengths of all samples were measured. Five days under treatment (DUT), shoot and root elongation lengths were determined, as well as visible phenotypes in response to salinity stress, using the modified standard evaluation system (SES) score developed by IRRI (IRRI 2013) to assess salinity tolerance in each accession (Additional file 2: Table S2). At 20 DUT, all phenotypes were scored again with the SES, and shoot and root elongation lengths were measured, after which the samples were collected. All plants were washed in tap water (twice) and then in distilled water twice to remove salts on the plant surface. Plants were separated into two parts (shoot and root), dried at 70 °C for 80 h, and weighed as shoot dry weight and root dry weight. The shoot dry weight of each accession was used for the genome-wide association study (GWAS).

### Mineral Analysis

To perform mineral analysis, 20 accessions with varying SES scores were selected from the initial 97 MDI accessions (Additional file 2: Supplementary Table S1). Plants grown in standard nutrient solution or with 100 mM NaCl for 20 days (20 DUT) were dried and divided into four parts: roots, upper stems (hereafter stems), lower stems, and leaves; lower stem samples were not analyzed due to contamination of the culture solution. Samples were ground to powder (30–40 mg) and digested with 2 mL of 60% (w/v) HNO_3_ in a tube at 107.5 °C in a DigiPREP apparatus (SCP Science, Quebec, Canada) for 4 h; 0.5 mL of 30% hydrogen peroxide was added, and the samples were heated to 107.5 °C. The tubes were allowed to cool to room temperature, and their volumes were adjusted to 10 mL with 2% (w/v) HNO_3_. The mineral ion composition for Na^+^, K^+^, Ca^2+^, and Mg^2+^ was determined by inductively coupled mass spectrometry (ICP-MS) (ELAN, DRC-e, Perkin-Elmer, Waltham, MA, USA) using a blank sample to account for chemical contaminations.

### Mineral Data Analysis

The average value of three biological replications for each accession was calculated. The relative elongation rates of shoots and roots were calculated as follows:

$${\text{R}} = \frac{{\text{A}}}{{\text{B}}}$$

where R is relative elongation, A is (shoot length/root length at 20 DUT—shoot length/root length at 0 DUT) of NaCl treatment, and B is (shoot length/root length at 20 DUT—shoot length/root length at 0 DUT) of control treatment.

### Genome-Wide Association Study (GWAS)

A dataset of 578,704 single nucleotide polymorphisms (SNPs) distributed across the 12 rice chromosomes in the 97 accessions previously obtained by restriction site–associated DNA sequencing (RAD-seq) (Tam et al. 2019) was filtered for a minimum call rate of 60%, a heterozygous rate below 20%, and a minor allele frequency below 2%, resulting in 37,643 SNPs. Imputation of missing genotypes was performed using Beagle v5.0 (Browning et al. 2018). The set of 37,643 SNPs was also filtered for 79 landraces based on the same criteria above, yielding 29,881 SNPs. GWAS was performed using a general linear model (GLM) in TASSEL 5.2.50 (Bradbury et al. 2007). The population structure component was determined using principal component analysis (PCA) implemented in TASSEL. The significant threshold was set to *p* < 0.0001 (− Log_10_ *p* value > 4.0). Subsequently, SNPs with a *q*-value (false discovery rate [FDR]-adjusted *p* value) lower than 0.05 were selected as significant markers with R software (Storey et al. 2004).

### Whole-Genome Resequencing

The whole-genome resequencing of the 20 MDI accessions selected from the 97 MDI accessions (Additional file 2: Table S1) was done using the Illumina Hiseq 150-bp Paired End platform, and 4.5 Gb data were obtained for each accession. After filtering and trimming low-quality reads, read mapping to reference genome Nipponbare and variant calling were performed using BWA-mem (Li and Durbin 2009) and the GATK pipeline (DePristo et al. 2011).

### Haplotype Analysis

Candidate genes (near peak SNPs) were searched in QTL and gene databases in the rice SNP seek database (Mansueto et al. 2017). The position of the candidate SNP was identified by RAP-DP (https://rapdb.dna.affrc.go.jp/). Linkage disequilibrium (LD) analysis was performed using SNPs located within 200-kb windows upstream and downstream of the SNP peaks based on the Haploview method according to Barrett et al. (2005).

## Results

### Evaluation of 97 Rice Accessions from the Mekong Delta Region

**Fig. 1:**
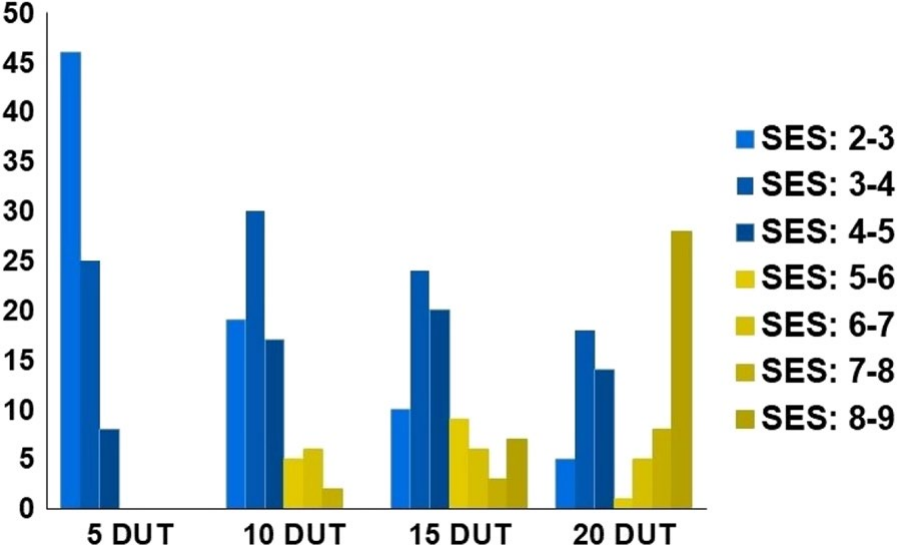
Changes in SES score distributions among 97 accessions from 5 to 20 days under treatment (DUT) with 100 mM NaCl. The responses of the 97 rice accessions to 100 mM NaCl treatment were assessed with three replications, evaluated by the SES score, and recorded at 5, 10, 15, and 20 DUT. As indicated in Additional file 2: Table S2, SES scores were grouped into seven classes: 2–3, 3–4, 4–5, 5–6, 6–7, 7–8, and 8–9, as indicated by different colors. SES scores of 2–5 defined salinity-tolerant accessions; SES score above 5 were considered salinity-sensitive accessions

We evaluated the salinity tolerance of 97 accessions from the Mekong Delta region to select highly tolerant accessions. In preliminary tests with 50 and 100 mM of NaCl solution, 100 mM NaCl clearly revealed differences across accessions (Additional file 2: Table S3). We scored the salinity responses according to SES developed by IRRI (Additional file 2: Table S2) (IRRI 2013). Distributions of SES scores for salinity-tolerance changed among different accessions at 5, 10, 15, and 20 days into salinity treatment (DUT). As the length of salinity exposure increased, so did the SES scores, with two peaks emerging with SES scores between 3–4 and 8–9 (Fig. 1). At 5 DUT, about 66% of all accessions presented a SES score below 3 (indicating high tolerance), while the remaining 34% of accessions had SES scores between 3 and 5 (tolerance). By contrast, only five accessions retained an SES score below 3 at 20 DUT, 47 accessions had an SES score of 3–5 (moderate tolerance), and 28 accessions had an SES score of 8–9 (sensitive) (Fig. 1). We concluded that 20 DUT is the most suitable screening condition to evaluate salinity responses of the accessions.

### Correlations Between Phenotypic Traits and SES Scores from 97 Accessions

**Fig. 2:**
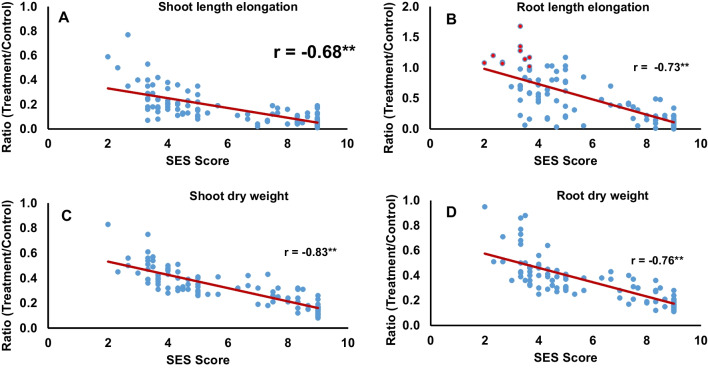
Correlations between four phenotypic traits and SES scores across the 97 accessions at 20 DUT with 100 mM NaCl. **A** Shoot length elongation ratio. **B** Root length elongation ratio. **C** Shoot dry weight ratio. **D** Root dry weight ratio. All correlations were significant (*p* value ≤ 0.01)*.* *r*, Pearson’s correlation coefficient

To explore possible correlations between SES scores and phenotypic traits, we examined shoot and root elongation lengths, as well as shoot and root dry weights after exposure to 100 mM NaCl for 20 days (Additional file 2: Table S4). Accordingly, we calculated the ratios of these four traits between NaCl-treated and control samples for all accessions. All ratios at 20 DUT were strongly and negatively correlated with SES scores (Fig. 2). Pearson’s correlation coefficients also supported these strong negative correlations, with values ranging from –0.68 to –0.83 (Fig. 2A–D). The shoot dry weight ratio showed the strongest correlation with SES score, with a Pearson’s correlation coefficient of –0.83 (Fig. 2C), while the shoot length ratio was least affected with a Pearson’s correlation coefficient of − 0.68 (Fig. 2A). When plotting the sum of the ratios for the four above traits as a function of SES score, we noticed that the top eight summed phenotypic values match eight of the nine lowest SES scores (Additional file 2: Table S4), indicating that SES scores are an acceptable index reflecting salinity tolerance. Among the examined accessions, MDI-21 showed the best performance in terms of salinity tolerance, as evidenced by a low SES score and high phenotypic ratios (Additional file 2: Table S4). Notably, MDI-21 was the variety Doc Phung, known to be highly salinity tolerant (Ho et al. 2018). Interestingly, according to our previous phylogenetic analyses of the MDI accessions (Tam et al. 2019), the closest accession to Doc Phung was MDI-13, called “Trang Phieu”, of which SES was 2.33, one of the most tolerant accessions (Additional file 2: Table S4).

### Mineral Concentrations in Saline Conditions

**Fig. 3:**
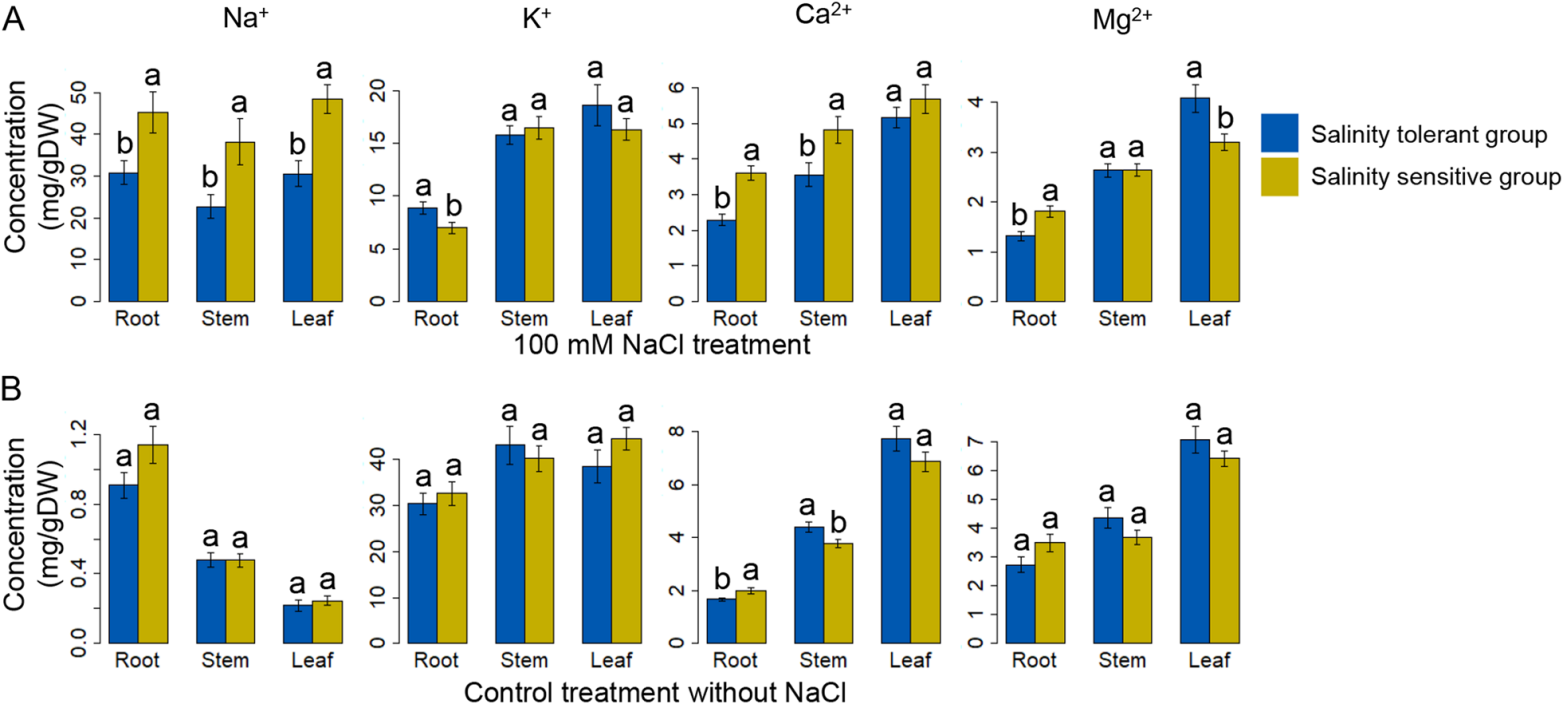
Concentrations of four mineral ions (Na^+^, K^+^, Ca^2+^, and Mg^2+^) in roots, stems, and leaves for salinity-tolerant/sensitive groups. Twenty accessions, consisting of eight tolerant (green: SES scores 2–4) and 12 sensitive (red: SES scores 4–9) accessions, were selected from the initial 97 accessions. **A** Accessions treated with 100 mM NaCl for 20 days (20 DUT). **B** Accessions grown under control conditions

We selected 20 accessions with varying levels of salinity response from the initial collection of 97 MDI landraces to examine their mineral concentrations. These accessions comprised eight salinity-tolerant accessions (SES < 4) and 12 sensitive accessions (SES > 4) (Additional file 2: Table S5). We determined the concentrations of four elements (Na^+^, K^+^, Ca^2+^, and Mg^2+^) in roots, stems, and leaves (Fig. 3, Additional file 2: Table S5). Under high-salinity conditions (100 mM NaCl), Na^+^ concentrations were significantly lower at *p-*value < 0.05 in the tolerant accessions compared to sensitive accessions in all three organs, as were Ca^2+^ concentrations in roots and stems (Fig. 3A). By contrast, Mg^2+^ concentrations were significantly higher in leaves of tolerant accessions than in those of sensitive accessions (Fig. 3A). We observed no differences between tolerant or sensitive accessions for K^+^ in leaves, Ca^2+^ in leaves, and Mg^2+^ in stems (Fig. 3A). Under normal growth conditions, the concentrations for Na^+^, K^+^, Ca^2+^, and Mg^2+^ in roots, stems, and leaves were not different between salinity-sensitive and -tolerant accessions (Fig. 3B). We tested for correlations between mineral concentrations and tolerance/sensitivity, which revealed that Na^+^ and Ca^2+^ concentrations are positively correlated with SES scores in the 20 selected accessions, in particular Na^+^ concentrations in leaves (*r* = 0.66) and Ca concentrations in roots (*r* = 0.765) and stems (*r* = 0.64) (Additional file 1: Fig. S2). We obtained the lowest mineral concentrations in MDI-21 (Doc Phung), which also had the lowest SES at 20 DUT (Additional file 2: Table S5). We observed no clear correlations between K^+^ concentrations and SES scores among the 20 accessions.

### Comparison of the OsHKT1;5 Gene Among 20 MDI Accessions and Nona Bokra/Pokkali

**Fig. 4:**
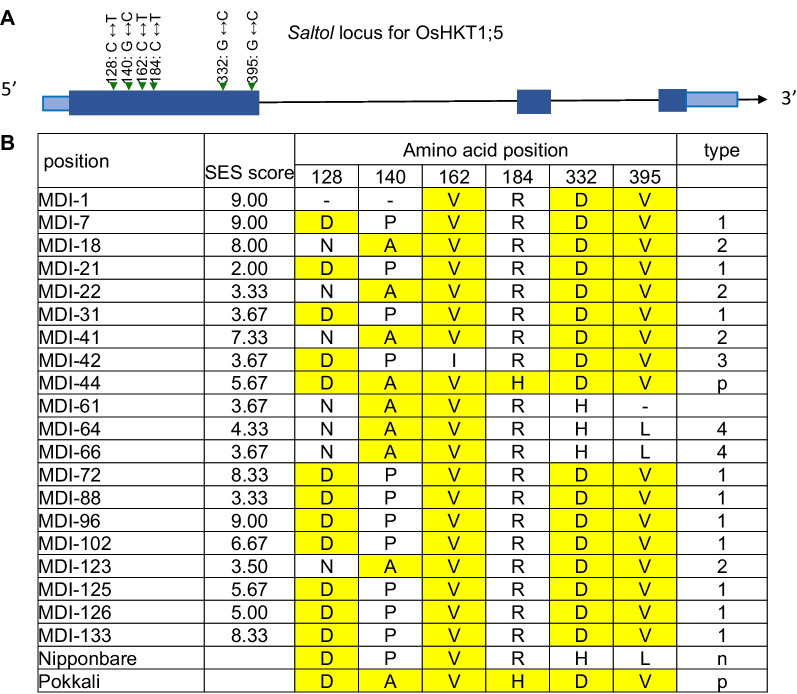
Comparison of amino acid sequences between 20 MDI accessions and Nona Bokra/Pokkali at OsHKT1;5 encoded by the *Saltol* locus. **A** Schematic diagram of the *OsHKT1;5* gene. Dark blue boxes, exons; light blue boxes, untranslated regions; lines, introns. The six positions of 20 MDI accessions indicate the polymorphisms leading to nonsynonymous amino acid (aa) changes relative to the *Saltol* allele of the Nona Bokra/Pokkali accessions. **B** Haplotype analysis of nonsynonymous amino acid substitutions at the six sites defined above in each of the 20 MDI accessions. Yellow and (−) represent shared amino acids with Nona Bokra/Pokkali and undetermined amino acids

The *OsHKT1;5* gene underlies the *Saltol* locus, whose alleles from the Nona Bokra and Pokkali varieties confer tolerance to high salinity. To assess whether *Saltol* contributes to salinity sensitivity/tolerance in the MDI accessions, we compared the *OsHKT1;5* genomic sequences from the 20 MDI accessions to the Nona Bokra/Pokkali allele. To this end, we extracted the *OsHKT1; 5* sequences and inferred the amino acid (aa) sequences from 20 MDI rice accessions representing 97 MDI rice accessions chosen for whole-genome resequencing. In Nona Bokra/Pokkali, the *OsHKT1;5* open reading frame was 1,662 bp in length and encoded a protein of 554 aa (Fig. 4A). Six amino acid residues showed nonsynonymous polymorphisms for OsHKT1;5 among the 20 MDI accessions, Nipponbare and Nona Bokra/Pokkali (Fig. 4B), defining six haplotypes. The accession MDI-44 shared the same *OsHKT1;5* haplotype with Nona Bokra/Pokkali, although it was not a highly tolerant accession, with an SES score of 4 (Fig. 4B). Haplotype 1 contained 10 accessions, including the highly tolerant MDI-21, and showed a broad range of SES scores (Fig. 4B). The four accessions belonging to haplotype 2 displayed varied SES scores, as with haplotype 1 (Fig. 4B). We concluded from these results that the genotype at *OsHKT1;5* is not strongly correlated with salinity tolerance of the 20 MDI accessions tested here. These results thus suggested that the salinity tolerance in the MDI accessions is genetically distinct from the *Saltol* locus harbored by Nona Bokra/Pokkali.

### Manhattan Plots for the SES Score of MDI Rice Accessions

**Fig. 5:**
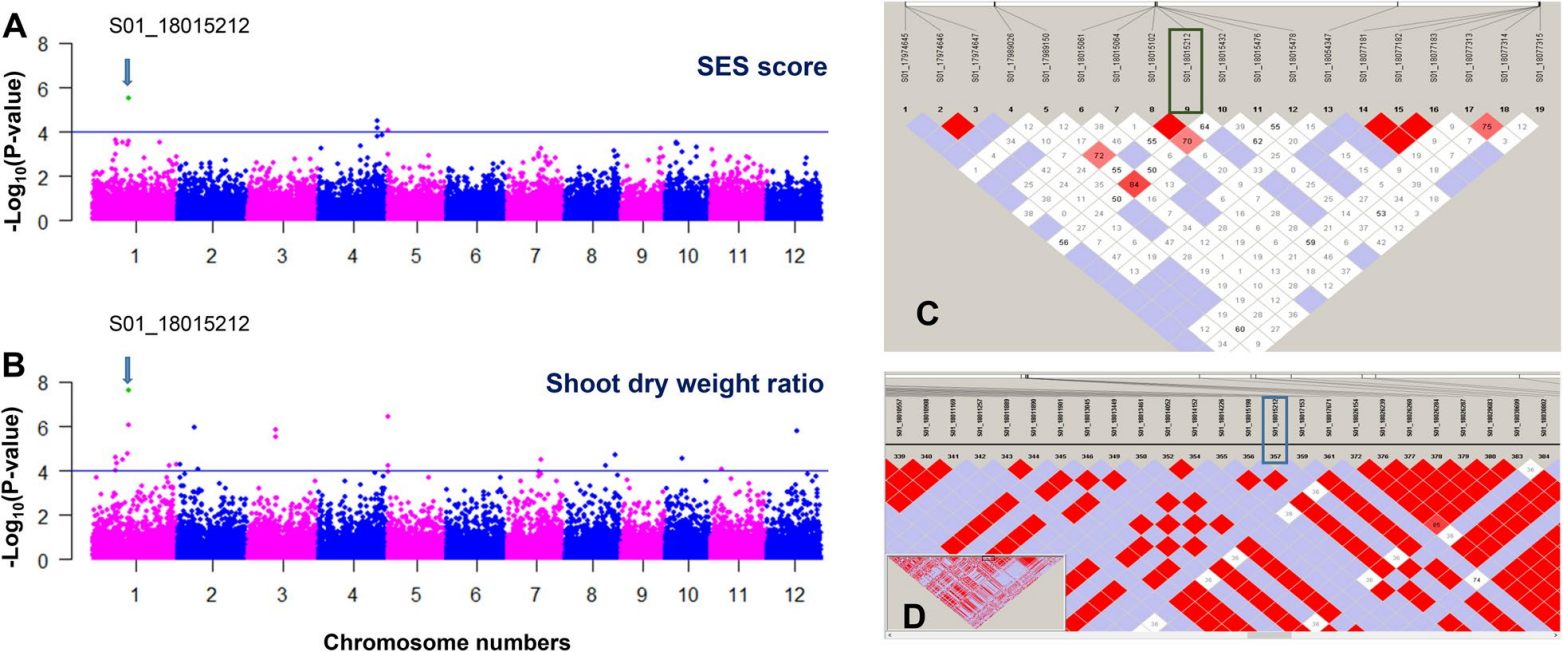
GWAS of salinity responses with SES scores and shoot dry weights for the MDI accessions. **A** Manhattan plot for SES scores with 29,881 imputed SNPs (Tam et al. 2019) from the 79 accessions at 20 DUT with 100 mM NaCl. **B** Manhattan plot for shoot dry weight ratio. Both analyses identified a single and identical SNP peak, S01_18015212, on chromosome 1. **C** Linkage disequilibrium (LD) analysis showing that S01_18015212 forms a single block using 19 SNPs within 200 kb obtained by RAD-seq analysis for the 79 accessions. **D** LD analysis using 657 SNPs from the 20 accessions, showing a single LD block within the 200-kb window

The above results showed that salinity tolerance in the MDI accessions may be regulated by loci different from the *Saltol* locus encoding the OsHKT1;5 transporter. To look for additional loci related to salinity tolerance, we performed GWASs using 37,643 imputed SNPs obtained from RAD-seq data from the 97 MDI accessions (Tam et al. 2019). We produced Manhattan plots using 29,881 SNPs from the 79 MDI landraces (97 MDI minus 18 improved varieties) using their SES scores at 20 DUT as the trait. We observed significant associations with three genomic regions for SES scores on chromosomes 1, 4, and 5. These three regions consisted of five SNP markers: one on chromosome 1, two on chromosome 4, and two on chromosome 5, with a significance threshold –Log (*p*-value) > 4.0 (Fig. 5A). When adjusting the threshold to account for a false discovery rate (*q*-value) < 0.1, only one SNP on chromosome 1 (S01_18015212) remained significant (Additional file 2: Table S6A).

We detected the same SNP peak when using shoot dry weight as the trait, which was the most highly correlated out of the four phenotypes measured, based on Pearson’s correlation coefficients, with SES scores (Fig. 5B). The GWAS returned 23 significant SNPs mapping to nine chromosomes for shoot dry weight with the threshold −Log (*p* value) ≥ 4.0, on chromosomes 1, 2, 3, 5, 7, 8, 10, 11, and 12 (Fig. 5B). We retained eight SNPs on five chromosomes with a *q*-value ≤ 0.05 (Fig. 5B). Importantly, SNP S01_18015212, which was associated with SES score, was the most significant for shoot dry weight (Additional file 2: Table S6B).

### Linkage Disequilibrium (LD) and Haplotypes Around the SNP Peak Region

We analyzed the SNPs over a 200-kb window (100 kb upstream and 100 kb downstream of the peak SNP). After filtering, we retained 19 SNPs for LD analysis based on the Haploview method (Fig. 5C, D). Surprisingly, the peak SNP (S01_18015212) did not group with other SNPs within the neighboring 200-kb window (Fig. 5C), indicating that no other nearby SNP interacts with SNP (S01_18015212). Furthermore, we examined SNP (S01_18015212) using whole-genome resequencing data from the 20 accessions, resulting in 657 SNPs within the same 200-kb window. Again, the peak SNP S01_18015212 was the only site showing a peak in Manhattan plots and showed no LD with other linked SNPs (Fig. 5D).

**Fig. 6:**
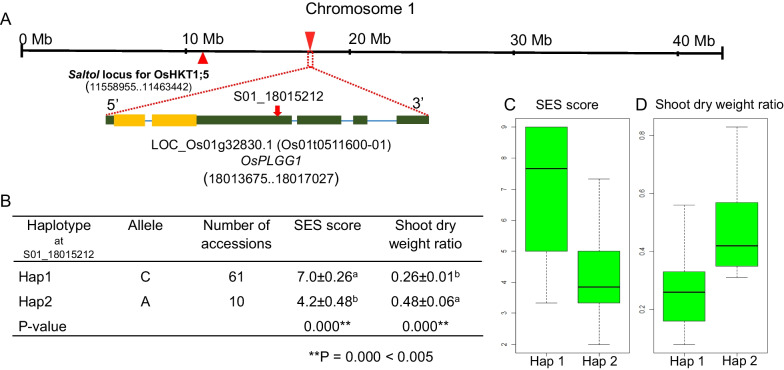
Location of the SNP peak on chromosome 1 and haplotype analysis. **A** Schematic representation of rice chromosome 1 and the position of SNP S01_18015212 within the 3′ UTR of LOC_Os01g3283 (*OsPLGG1*). This gene is 6.5 Mb away from the *Saltol* locus. According to RAP-DP, translated and untranslated regions in LOC_Os01g3283 were indicated by orange and green rectangles, respectively. ORF **B** S01_18015212 defines two haplotypes among 71 accessions: Hap 1 was found in 61 accessions with nucleotide C, and Hap 2 was found in 10 accessions with **A**. The remaining eight accessions had no exact haplotype (heterozygous alleles). **C**, **D** Significant differences in SES scores (**C**) and shoot dry weight ratio (**D**) values between Hap 1 and Hap 2 accessions

SNP S01_18015212 mapped to the 3′ untranslated region (UTR) of LOC_Os01g32830 on chromosome 1, about 6.5 Mb away from the *Saltol* locus (Fig. 6A), clearly indicating that the *Saltol* allele does not play a role in salinity-tolerance in these accessions. LOC_Os01g32830 encodes a predicted chloroplast-localized transmembrane protein: chloroplast glycolate/glycerate translocator 1 (OsPLGG1) (Shim et al. 2019). PLGG1 transports glycolate/glycerate produced in chloroplasts to peroxisomes during photorespiration (Pick et al. 2013).

We identified 3 possible haplotypes among 79 rice accessions at SNP S01_18015212: 61 accessions harboring the C allele (Hap 1), 10 carrying the A allele (Hap 2), and 8 heterozygous accessions (not evaluated here for haplotype comparison) (Fig. 6B). The average SES score (4.2 ± 0.48) of accessions with the A allele was significantly lower than in accessions with the C allele (7.0 ± 0.26) (Fig. 6B, C). The shoot dry weight ratio showed the opposite pattern relative to SES scores, with Hap1 associated with a lower ratio than Hap 2 (Fig. 6B, D). We concluded that Hap 2 is a salinity-tolerant haplotype, while Hap 1 was sensitive to salinity. The variety Doc Phung belonged to Hap 2.

## Discussion

### Geographical Association with Salinity Stress Responses in MDI Strains

**Fig. 7:**
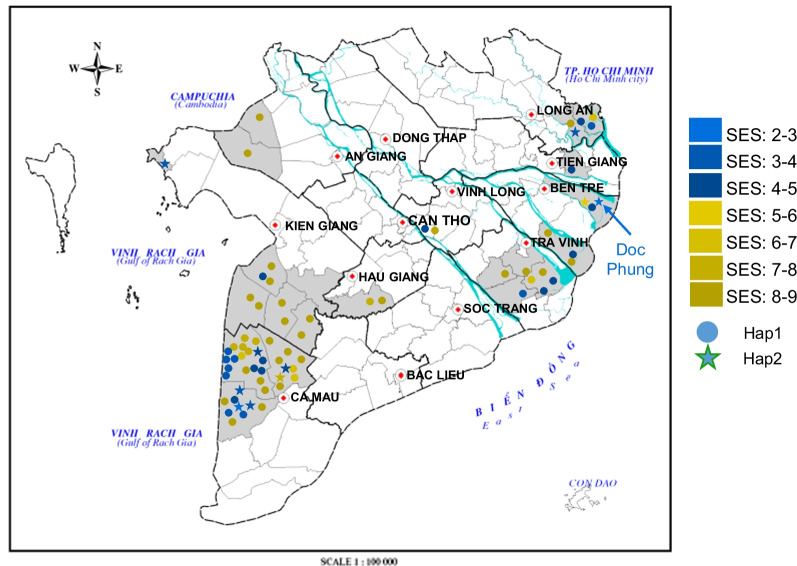
Geographical distribution of the 71 MDI landrace accessions in the Mekong Delta as a function of their S01_18015212 haplotype. The map shows the Mekong Delta region in Vietnam. Different colors indicate varying SES scores from Fig. 1. The circles and stars indicate Hap 1 (61 accessions) and Hap 2 (10 accessions), respectively

The evaluation of SES scores for 97 MDI accessions exposed to 100 mM NaCl for 20 days revealed two major peaks for salinity-tolerance: one peak from a group with high-salinity-tolerance (SES score between 3 and 5) and another peak from a group with low-salinity-tolerance (SES scores between 8 and 9) (Fig. 1). We observed regional differences between the coastal and inland areas of the Mekong Delta for the distribution of high- and low-salinity-tolerance groups, especially for high-salinity-tolerant accessions, which were mostly derived from the coastal Ca Mau region (Fig. 7, Additional file 2: Table S1). It is possible that adaptive selection for salinity-tolerance acted on these accessions originating from the Ca Mau coastal region, in an analogous manner to the salinity-tolerant varieties Nona Bokra and Pokkali, whose indigenous varieties originated from Bangladesh and coastal India, respectively (Emon et al. 2015; Gregorio and Senadhira 1993). On the other hand, Doc Phung does not originate from the Ca Mau region but from the Ben Tre region, and the salinity-tolerant allele of the Hap2 type is widely distributed in the coastal areas of the Mekong Delta (Fig. 7). In agreement, inland strains tended to be less salinity tolerant, may have grown with little exposure to salinity stress, and were likely to possess genomic structures similar to accessions from Cambodia and Laos (Tam et al. 2019). The three genomic regions identified by GWASs clearly illustrated the difference in genome structure between local coastal and inland accessions of the Mekong Delta (Fig. 7). The observed difference in salinity-tolerance, along with the differences in genomic structures, suggests that genetic exchange between the native lineages has been limited.

### Genetic Association with Diversity of Salinity Stress Responses in MDI Strains

We used the SES score for salinity-tolerance developed by IRRI to evaluate our accessions at the seedling stage (IRRI 2013). SES scores for each accession were strongly and negatively correlated with their salinity response phenotypes and the ratios for shoot length, root length, shoot dry weight, and root dry weight, with Pearson’s correlation coefficients no higher than –0.67 (Fig. 2). These correlations demonstrated that the SES score is a good indicator of salinity-tolerance. Shoot dry weight ratios showed the strongest correlation with SES scores (Fig. 2). Using the shoot dry weight ratio and SES scores for GWAS, we identified a significant peak at a single SNP on chromosome 1 for both traits (Fig. 5A, B). We identified the same single SNP, S01_18015212, in a subsequent GWAS based on the resequencing data of 20 accessions with different SES scores (Fig. 5C, D). Although several salinity-response-related genes, including the *Saltol* locus (Ren et al. 2005; Thomson et al. 2010), are located on chromosome 1, the gene to which the peak SNP mapped is a previously uncharacterized gene, with the SNP located in the 3′ UTR (Fig. 6A).

### Possible Functions of the SNP in the 3′ UTR of LOC_Os01g32830 (OsPLGG1)

The IRRI Rice SNP-Seek Database (Mansueto et al. 2017) and RAP DB (Sakai et al. 2013) revealed that SNP S01_18015212 maps to LOC_Os01g32830 (Os01t0511600-01). Os01t0511600-01 encodes OsPLGG1 responsible for the transport of glycolate/glycerate from chloroplasts to peroxisomes (Shim et al. 2019). Mutants in *OsPLGG1* exhibit lower photosynthetic efficiency, starch accumulation, plant height, and crop productivity (Shim et al. 2019). Salinity stress is known to result in excess reactive oxygen species (ROS), which interferes with normal photosynthesis and negatively affect plant growth (Pawlowicz et al. 2018; Yang et al. 2020). We hypothesize that variation in OsPLGG1 activity in transporting glycolate/glycerate from chloroplasts to peroxisomes may contribute to the accumulation of ROS in response to salinity stress, which affects photosynthetic reactions and plant growth.

SNP S01_18015212 is located in the 3′ UTR of the gene, which typically participates in the regulation of mRNA stability, localization, or translation; this SNP may thus affect protein abundance (Sun et al. 2017). In addition, 3′ UTRs are known to control gene expression in plants, especially when exposed to environmental stress (Srivastava et al. 2018). To validate our prediction, the genome editing experiment will provide useful information on the relationships between salinity response and function of the candidate gene.

### Physiological Characteristics of Distinct Salinity Tolerance in the MDR Landraces

In general, excess Na^+^ decreases Ca^2+^ uptake, while supplemental Ca^2+^ increases Ca^2+^ concentrations in plants, resulting in improved plant growth under salinity stress conditions (Cramer et al. 1991). To precisely evaluate the correlation between accessions tolerant or sensitive to salinity in this study, we analyzed the salinity response of 20 MDI accessions from the Vietnamese Mekong Delta (Fig. 3). The salinity response was highly variable among 20 MDI accessions, which can be divided into two distinct groups: a tolerant group of eight accessions (SES score < 4) and a sensitive group of 12 accessions (SES score > 4) (Additional file 2: Table S4). We also determined mineral concentrations for roots, leaves, and upper stems in these 20 accessions (Additional file 2: Table S5, Additional file 1: Fig. S2). Concentrations for Na^+^, Ca^2+^, the Na^+^/K^+^ ratio, and the Na^+^/Ca^2+^ ratio in the tolerant group were lower than those obtained for the sensitive group (Fig. 3A), which was consistent with the results reported by Kumar et al. (2008), except for Ca^2+^ concentrations, where Kumar et al. (2008) detected higher Ca^2+^ concentrations in the tolerant group relative to the sensitive group. Moreover, SNP S01_18015212 is located within the mapping interval of three QTLs for salinity tolerance (QTAROqtl-195, QTAROqtl-196, and QTAROqtl-197), which function in Na^+^ uptake, K^+^ concentrations, and the Na^+^/K^+^ ratio, respectively (Mansueto et al. 2017).

In roots, Ca^2+^ accumulates mainly in the cell wall (Demarty et al. 1984) by binding to the cation exchange sites of root cell wall pectin. Plants with low root Ca^2+^ concentrations thus tend to exhibit low cation exchange capacity (CEC) in their root cell walls (Marschner 2012). Similarly, Na^+^ binds to root cell wall pectin and causes cell wall damage (Byrt et al. 2018). Therefore, crops with low root CEC have been reported to be more tolerant to salinity than those with high root CEC (Bajwa and Bhumbla 1971). The positive correlations observed between SES scores and both Na^+^ and Ca^2+^ concentrations in roots in this study (Additional file 1: Fig. S2) suggest that root CEC is smaller in the tolerant accessions including Doc Phung. Compared to the salinity-sensitive accessions, the salinity-tolerant strains of the Mekong Delta population, represented by Doc Phung, may present lower cell wall damage due to lower Na^+^ and Ca^2+^ binding capacity to cell wall pectin (Byrt et al. 2018; Marschner 2012). Future exploration of the association between limited Na^+^ and Ca^2+^ binding to cell wall pectin and the SNP located in the 3′ UTR of *OsPLGG1* will be useful to study the mechanism of salinity-tolerance in Doc Phung.

### Genetic Specificity and Origin of Doc Phung

Several articles have reported that Doc Phung shows superior salinity-tolerance, and this study clearly validated the evidence for this promising salinity-tolerant accession (Ho et al. 2018; Le 1999; Tin et al. 2021). First, SES evaluation showed that Doc Phung has the highest salinity-tolerance and the highest values for shoot length ratio, shoot dry weight ratio, and root dry weight ratio, all highly correlated with salinity-tolerance (Fig. 2). GWASs based on these trait indices among the MDI accessions showed that a promising SNP associated with salinity-tolerance maps to chromosome 1 (Fig. 6), in a genomic location distinct from that of the *Saltol* locus encoding the HKT1;5 transporter, which is associated with salinity-tolerance in the varieties Nona Bokra and Pokkali (Fig. 6). The *HKT1;5* genotype at the *Saltol* locus in Doc Phung was different from the allele harbored by Nona Bokra and Pokkali (Fig. 4). Therefore, the origin of salinity-tolerance in Doc Phung may have originated from accessions cultivated in the Mekong Delta or from the coastal periphery. SNP S01_1801521 in the 3′ UTR of *OsPLGG1* provides a new option for salinity-tolerant alleles in rice breeding. It is necessary to analyze the relationship between this gene and salinity-tolerance. This SNP is useful for stacking multiple salinity tolerance alleles in rice breeding.

## Conclusion

We analyzed the relationship between the degree of salinity-tolerance and physiological characteristics and performed GWASs for the Mekong Delta rice population. We focused on the landrace variety Doc Phung and confirmed it to be particularly resistant to salinity exposure (Ho et al. 2018; Le 1999; Tin et al. 2021). The Na^+^ and Ca^2+^ concentrations in the salinity-tolerant accessions allowed us to propose a salinity-tolerant mechanism caused by smaller root CEC. In addition, we identified a gene with a SNP specific to salinity-tolerant accessions obtained from GWASs. This SNP was located in the 3′ UTR of *OsPLGG1*. Overall, our results provide evidence that the salinity-tolerant accessions in the Mekong Delta population, such as Doc Phung, have a different *Saltol* allele from that of Nona Bokra and Pokkali. The causative genomic region of salinity-tolerance in Doc Phung, which might have been adapted to the Mekong Delta, is a new source for rice salinity tolerance. This study will provide a concrete countermeasure for rice cultivation against the salinity intrusion in the coastal areas of the Mekong Delta due to climate change.

## Supplementary Information

**Additional file 1: Figure S1.** MDI accessions during the salinity tolerance test. Rice seedlings were grown hydroponically in a 40-L container with floating foam material in the greenhouse. **Figure S2.** Correlations between SES scores at 20 DUT and mineral concentrations across the 20 MDI accessions. Mineral concentrations and SES scores are plotted for four mineral ions (Na+, K+, Ca2+, and Mg2+) in roots, stems, and leaves. The 20 accessions were treated with 100 mM NaCl for 20 days (20 DUT). r, Pearson’s correlation coefficient.

**Additional file 2: Table S1.** List of Mekong Delta rice accessions examined in this study. **Table S2.** Phenotypic features for the standard evaluation system (SES) scores. **Table S3.** Preliminary test for salinity-tolerant/sensitive groups with 50 and 100 mM NaCl. **Table S4.** SES scores and phenotypic values in the 97 MDI accessions and their rankings. **Table S5.** Concentrations (mg/gDW) for mineral ions (Na+, K+, Ca2+, and Mg2+) and Na2+/K+ ratio in roots, stems, and leaves of 20 accessions exposed to 100 mM NaCl for 20 days (20 DUT) and control samples. **Table S6A.** SNPs above the threshold of p-value at 1.00E–04 in a Manhattan plot for SES score at 20 DUT (Fig. 5A). **Table S6B.** SNPs above the threshold of p-value at 1.00E–04 in a Manhattan plot for shoot dry weight ratio (Fig. 5B).

## Data Availability

The RAD-seq data set of the 97-accession MDI has been deposited in the DDBJ database (accession number DRA008414). The data set of the whole-genome resequencing of the 20 MDI accessions has been registered as SUB10778038 into Sequence Read Archive (SRA). The request of availability of the accessions used in this study is asked to TTM (ngttam@ctu.edu.vn).

## Abbreviations

MDI: Mekong Delta Development Research Institute
SES: The standard evaluation system
CEC: Cation exchange capacity
GWAS: Genome-wide association study
*OsPLGG1*: Chloroplast glycolate/glycerate translocator 1
HKT1: HIGH-AFFINITY K^+^ TRANSPORTER1
IRRI: The International Rice Research Institute
DAT: Days after transfer
DUT: Days under treatment
SNP: Single nucleotide polymorphism
RAD-seq: Restriction site–associated DNA sequencing
GLM: General linear model
PCA: Principal component analysis
LD: Linkage disequilibrium
aa: The amino acid
