# Supplementary material for: Identification of a Saltol-Independent Salinity Tolerance Polymorphism in Rice Mekong Delta Landraces and Characterization of a Promising Line, Doc Phung
Source: Rice (N Y). 2022 Dec 18;15:65. doi: 10.1186/s12284-022-00613-0 (PMC9760585; doi:10.1186/s12284-022-00613-0)
Supplement: Supplementary file 1 — Additional file 1: Figure S1. MDI accessions during the salinity tolerance test. Rice seedlings were grown hydroponically in a 40-L container with floating foam material in the greenhouse. Figure S2. Correlations between SES scores at 20 DUT and mineral concentrations across the 20 MDI accessions. Mineral concentrations and SES scores are plotted for four mineral ions (Na+, K+, Ca2+, and Mg2+) in roots, stems, and leaves. The 20 accessions were treated with 100 mM NaCl for 20 days (20 DUT). r, Pearson’s correlation coefficient. [file 12284_2022_613_MOESM1_ESM.pptx]

## Slide 1
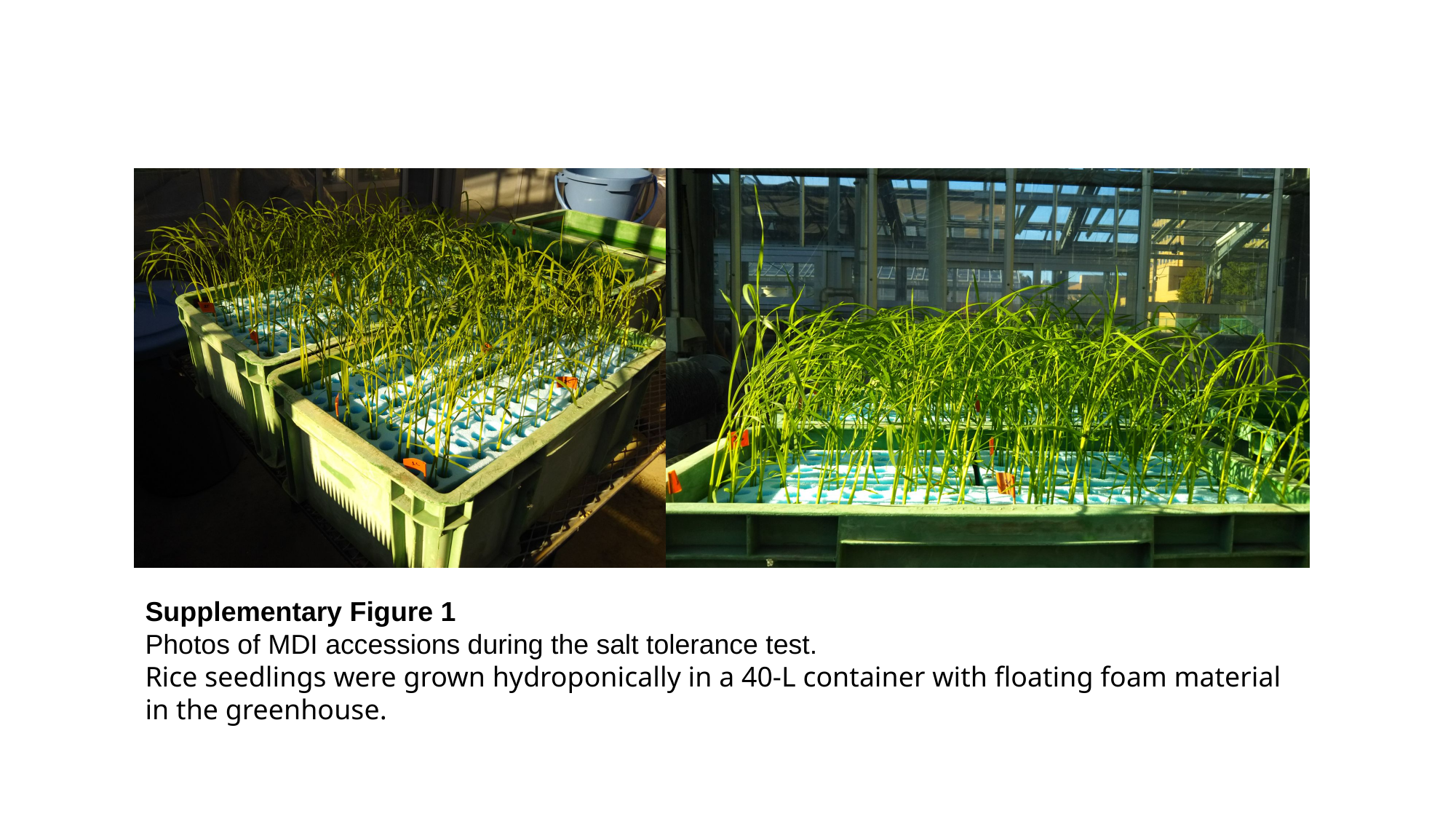

Supplementary Figure 1
Photos of MDI accessions during the salt tolerance test.
Rice seedlings were grown hydroponically in a 40-L container with floating foam material in the greenhouse.

## Slide 2
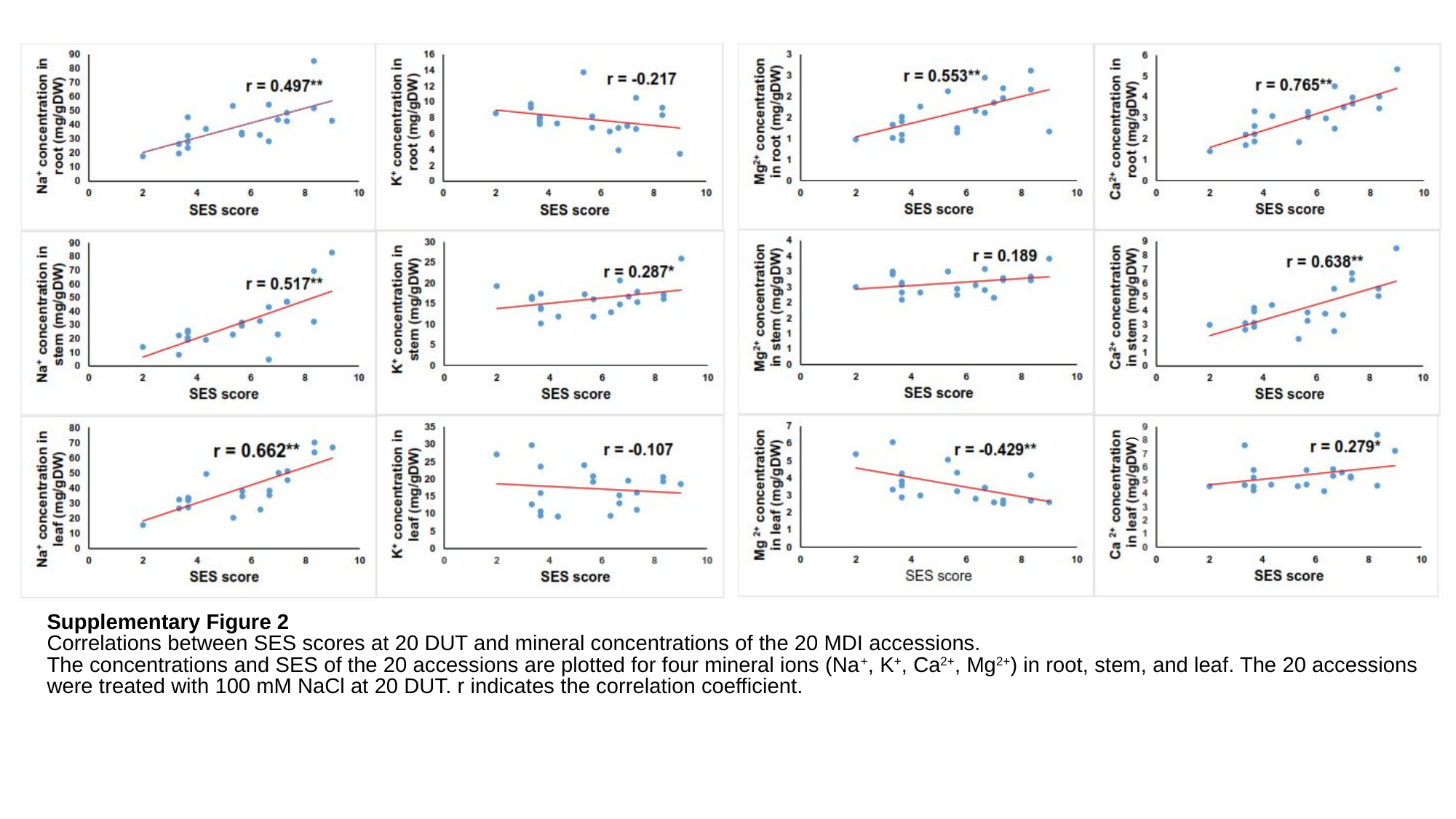

)
# Supplementary Figure 2 Correlations between SES scores at 20 DUT and mineral concentrations of the 20 MDI accessions. The concentrations and SES of the 20 accessions are plotted for four mineral ions (Na+, K+, Ca2+, Mg2+) in root, stem, and leaf. The 20 accessions were treated with 100 mM NaCl at 20 DUT. r indicates the correlation coefficient.
